# 

**Published:** 1858-05

**Authors:** 


### LIST OF COLLABORATORS.

S. G. Armor, M.D., Professor of Pathology and Clinical Medicine, Missouri Med. College.
John L. Atlee, M.D., President Pennsylvania State Medical Society.
Walter F. Atlee, M.D., Philadelphia.
John Bell, M.D., Philadelphia, formerly Professor of Medicine, Medical College of Ohio.
T. S. Bell, M.D., Professor of Medicine, University of Louisville.
S. M. Bemiss, M.D., Louisville, Ky.
L. P. Blackburn, M.D., Natchez, Miss.
George C. Blackie, M.D., Professor of Botany, University of Nashville.
G. C. Blackman, M.D., Professor of Surgery, Medical College of Ohio.
W. S. Bowen, M.D., New York.
J. L. Bobbs, M.D., formerly Professor of Anatomy, Indianapolis.
W. M. Boling, M.D., Montgomery, Ala., formerly Professor of Midwifery, Transylvania University, Lexington, Ky.
N. Bozeman, M.D., Montgomery, Ala.
J. T. Bradford, M.D., Augusta, Ky.
John H. Brinton, M.D., Lecturer on Operative Surgery, Philadelphia.
W. S. Chipley, M.D., Professor of Medicine, Transylvania University.
A. Clapp, M.D., New Albany, Ia.
D. B. Cliffe, M.D., Franklin, Tenn.
J. Da Costa, M.D., Lecturer on the Practice of Medicine at the Philad. Med. Association.
M. Dupierris, M.D., Havana, Cuba.
W. F. Edgar, M.D., United States Army.
S. C. Farrar, M.D., Jackson, Miss.
C. S. Fenner, M.D., Memphis, Tenn.
Austin Flint, M.D., Professor of Clinical Medicine and Pathology, University of Buffalo.
Charles Frick, M.D., Baltimore.
W. Y. Gadbury, M.D., Benton, Miss.
O. C. Gibbs, M.D., Chautauque County, New York.
Dr. J. P. Hall, Glasgow, Ky.
John Hardin, M.D., Professor of Obstetrics, Kentucky School of Medicine, Louisville.

EDWARD HARTSHORNE, M.D., One of the Surgeons to Wills Hospital for Diseases of the Eye, Philadelphia.

E. B. HASKINS, M.D., Clarksville, Tenn., late President of the Tennessee State Medical Society.

C. A. HENTZ, M.D., Marianna, Florida.

ADDINELL HEWSON, M.D., One of the Surgeons to Wills Hospital for Diseases of the Eye, Philadelphia.

SAMUEL L. HOLLINGSWORTH, M.D., late Editor of the Medical Examiner, Philadelphia.

WILLIAM A. HAMMOND, M.D., United States Army.

WILSON JEWELL, M.D., Philadelphia, President of the Quarantine and Sanitary Convention.

G. A. KUNKLER, M.D., Madison, Ia.

WM. V. KEATING, M.D., Lecturer on Obstetrics and the Diseases of Women, in the Philadelphia Medical Association.

BEN. LOGAN, M.D., Shreveport, La.

A. LOPEZ, M.D., Mobile, Ala., formerly President of the Alabama State Medical Society.

GEORGE R. MOREHOUSE, M.D., Philadelphia.

S. WEIR MITCHELL, M.D., Lecturer on Physiology at the Philad. Med. Association.

B. I. RAPHAEL, M.D., New York.

J. C. REEVE, M.D., Dayton, Ohio.

L. C. RIVES, M.D., formerly Professor of Midwifery, Medical College of Ohio.

J. LAWRENCE SMITH, M.D., Professor of Chemistry, University of Louisville.

W. C. SNEED, M.D., Frankfort, Ky., President Kentucky State Medical Society.

C. H. SPILMAN, M.D., Harrodsburg, Ky., late President of Kentucky State Medical Society.

GEO. SUTTON, M.D., Aurora, Ia.

W. L. SUTTON, M.D., Georgetown, Ky., formerly President Kentucky State Medical Society.

HORATIO R. STORER, M.D., formerly one of the Physicians to the Boston Lying-in Hospital, Boston, Mass.

S. THOMPSON, M.D., Albion, Ill.

E. WILLIAMS, M.D., Cincinnati, Ohio.

### Bi-Monthly Bibliographical Record.

AMERICAN.

The Institutes of Medicine. By Martyn Paine, M.D., Professor of the Institutes of Medicine and Materia Medica in the University of New York, &c. Fourth edition. 8vo., pp. 1095. Harper & Brothers: New York, 1858. (From the publishers.)

The Dispensatory of the United States of America. By George B. Wood, M.D., and Franklin Bache, M.D. Eleventh edition. Carefully revised. 8vo., pp. 1583. J. B. Lippincott & Co.: Philadelphia, 1858. (From the publishers.)

Plates to Wilson on the Skin. Fourth edition. Blanchard & Lea: Philadelphia, 1857. (From the publishers.)

Dysentery; Its Pathology and Treatment. Clinical Lectures delivered at the Jackson Street Hospital. By Robert Campbell, M.D., &c. Pamphlet, pp. 65. 1858. (From the author.)

Elements of Inorganic Chemistry. By Thomas Graham, F.R.S. L.& E., late Professor of Chemistry in University College, London. Second American from the second revised and enlarged London edition. 8vo., pp. 852. Blanchard & Lea: Philadelphia, 1858. (From the publishers.)

Researches on Primary Pathology and the Origin and Laws of Epidemics. By M. L. Knapp, M.D. Vol. ii. 8vo., pp. 336. Robb, Pile & McElroy: Philadelphia, 1858.

FOREIGN.

On Consumption; Its Nature, Symptoms, and Treatment. By Richard Payne Cotton, M.D. Second edition. 8vo., pp. 302. London, 1858.

Das Wasser Der Neuven. In Physiologischer und Pathologische Beziechung. Par G. Birkner. 8vo. Augsburg, Rieger, 1858.

Diseases of the Rectum. By Richard Quain, F.R.S. Second edition. 12mo. London, 1857.

A Guide to the Treatment of Diseases of the Skin. By Thomas Hunt, F.R.C.S. Second edition. Fcap. 8vo., pp. 212. London, 1857.

The Successful Treatment of Scarlet Fever. Also Observations on the Crowing Inspiration of Infants. By P. Hood. pp. 200. London, 1857.

Stricture of the Urethra; Its Pathology and Treatment. By Henry Thompson, F.R.C.S. 8vo. London, 1857.

On Nature and Art in the Cure of Disease. By Sir John Forbes, M.D. 8vo. London, 1858.

The Ganglionic Nervous System; Its Structure, Functions, and Diseases. By James George Davey, M.D. 8vo. London, 1858.

Kinder-Diätetik. Von L. W. Mauthner Ritter von Mautstein. Dritte veränderte und vermehrte auflage. 8vo., pp. 272. Wien, 1857.

Rheumatism; Its Nature, Causes, and Cure. Gout; Its Nature, Causes, Cure, and Prevention. By James Alexander, M.D. 8vo. London, 1858.

On the Diseases of the Bladder and Prostate Gland. By William Coulson. Fifth edition. 8vo. London, 1857.

The Diseases of Children. By Fleetwood Churchill, M.D. Second edition. 8vo., pp. 782. Dublin, 1858.

Anteckningar till Föreläsningar i Patologisk Anatomi, hóllne vid Karolinska Mediko-Kirurgiska Institutet. Af Gustaf Von Düben. Första Häftet. Stockholm, 1857. 8vo., pp. 127.

Prone and Postural Respiration in Drowning and other Forms of Apnoea or Suspended Respiration. By (the late) Marshall Hall, M.D. 8vo., pp. 216. London, 1857.

Dislocations and Fractures. By Joseph Maclise, F.R.C.S. Fasciculus 1. London, 1858.

Capacité Vitale du Poumon, les Rapport Physiologiques et Pathalogiques avec les Maladies de la Poitrine. Par le Docteur B. Schnepf. 8vo., pp. 515. Paris, 1858.

Leçons de Toxicologie. Par L. Orfila. 8vo., pp. 120. Paris, 1857.

Handbuch der Praktischen Chirurgie für Arzte und Wundärzte. Von Dr. Victor Bruns, Professor der Chirurgie und Chirurgischen Klinik in Tübingen. Zweite Abtheilung. Rau und Geschmacksorgan. 8vo., pp. 544. Tübingen, 1857.

